# The Impact of Structural Inequities on Older Asian Americans During COVID-19

**DOI:** 10.3389/fpubh.2021.690014

**Published:** 2021-08-16

**Authors:** Kris Pui Kwan Ma, Adrian Matias Bacong, Simona C. Kwon, Stella S. Yi, Lan N. Ðoàn

**Affiliations:** ^1^Department of Family Medicine, University of Washington School of Medicine, Seattle, WA, United States; ^2^Department of Community Health Sciences, Fielding School of Public Health, University of California, Los Angeles, Los Angeles, CA, United States; ^3^Department of Population Health, Section for Health Equity, New York University Grossman School of Medicine, New York, NY, United States

**Keywords:** COVID-19, Asian Americans, racism, intersectionality, aging, older adults

## Abstract

Structural racism manifests as an historical and continued invisibility of Asian Americans, whose experiences of disparities and diverse needs are omitted in research, data, and policy. During the pandemic, this invisibility intersects with rising anti-Asian violence and other persistent structural inequities that contribute to higher COVID-19 mortality in older Asian Americans compared to non-Hispanic whites. This perspective describes how structural inequities in social determinants of health—namely immigration, language and telehealth access, and economic conditions—lead to increased COVID-19 mortality and barriers to care among older Asian Americans. Specifically, we discuss how the historically racialized immigration system has patterned older Asian immigrant subpopulations into working in frontline essential occupations with high COVID-19 exposure. The threat of “public charge” rule has also prevented Asian immigrants from receiving eligible public assistance including COVID-19 testing and vaccination programs. We highlight the language diversity among older Asian Americans and how language access remains unaddressed in clinical and non-clinical services and creates barriers to routine and COVID-19 related care, particularly in geographic regions with small Asian American populations. We discuss the economic insecurity of older Asian immigrants and how co-residence in multigenerational homes has exposed them to greater risk of coronavirus transmission. Using an intersectionality-informed approach to address structural inequities, we recommend the disaggregation of racial/ethnic data, meaningful inclusion of older Asian Americans in research and policy, and equitable investment in community and multi-sectoral partnerships to improve health and wellbeing of older Asian Americans.

## Introduction

The disproportionate impact of COVID-19 among racially and ethnically diverse older adults has shed light on the persistent inequities against these marginalized populations (1). Asian Americans aged 45 years and older had higher COVID-19 attributable mortality compared to non-Hispanic whites (2), and Asian Americans had 35% more deaths in 2020 than their average for the last 5 years, the second-highest percent increase in excess deaths from COVID-19 and other causes (3). Older age and the presence of underlying chronic illnesses increases the risk of hospitalization and mortality from COVID-19 infection (4). However, age alone cannot fully explain COVID-19-related health disparities and the mortality gap, or the pre-pandemic health disadvantages, between minoritized older adults and their white counterparts (5, 6). Instead, the confluence of structural inequities amplifies the invisibility and exclusion of older Asian Americans in research and policy and contribute to the differential outcomes and unequal impact of COVID-19.

Emerging research literature has demonstrated that structural inequalities underlie COVID-19 disparities among Black and Latinx older adults (7, 8) but few have included older Asian Americans (6). Intersecting social processes and structures distinguish the older Asian American COVID-19 experience from younger Asian Americans, other minoritized populations, and by Asian American subgroup (9). We describe how structural inequities exacerbate existing vulnerabilities of older Asian Americans and make recommendations, using an intersectionality approach, to address the unequal brunt of COVID-19 on older Asian Americans communities (9).

## Invisibility, Masked Heterogeneity, and Scapegoating of Asian Americans

Asian Americans are the fastest growing racial/ethnic group in the general population and older adult population aged 65 years and above in the United States (U.S.) (10, 11). Structural racism for Asian Americans manifests as historical and continued invisibility of their health and service disparities in scientific research, health data, and public policy (12, 13). Asian American narratives and needs remain ignored in decision-making, with insufficient resources to address the longstanding health disparities that have worsened during the pandemic. The invisibility of Asian Americans and continued masked heterogeneity of Asian subgroups is reinforced by the lack of standardized racial/ethnic data collection, which is a manifestation of structural inequity in public health surveillance. In practice, this means there is a paucity of health data on older Asian Americans, and data that are further disaggregated by ethnic subgroups (13, 14). Public health surveillance systems have been intentionally designed to mask disparities in health and healthcare use among Asian subgroups compared to the broader Asian American group and how health vary within each Asian subgroup (14). Without meaningful data collection of Asian American subgroups, there remains a poor evidence-base to demand action from policymakers and research priorities to address the inequitable distribution of health risks and outcomes (12).

The model minority is the stereotype that Asian Americans have achieved educational and economic success relative to other racial/ethnic minority groups and has created a false perception that Asian Americans do not need help, when in fact disaggregated data demonstrate the disproportionate COVID-19 impact on Asian Americans, with variation by Asian subgroup and demographic characteristics (15). For example, South Asians had the highest COVID-19 infection rates and Chinese Americans had the greatest mortality of all Asian groups (16). Vietnamese Americans with hypertension and who worked in high-contact industries and South Asians with diabetes and who worked in healthcare/gig economy were are increased risk of infection (17).

Asian Americans have been blamed for the pandemic (18). A survey from June 2020 reported that 31% of Asian Americans had experienced racial/ethnic slurs or jokes since the beginning of the pandemic, compared to Black (21%), Latinx (15%), and white (8%) adults (19). Between March 2020 to March 2021, Stop AAPI Hate received 6,603 anti-Asian hate incident reports (i.e., verbal harassment, shunning, physical assault), with the greatest reporting among Chinese, Korean, Filipino and Vietnamese adults, and 7% of reports were from Asians aged 60 years and older (20). We suspect the number of hate incidents are underreported due to digital access and literacy to report and general fears of reporting due to retribution and immigration status. The spike in anti-Asian discrimination negatively impacted the physical and mental health of Asian Americans and revealed the structural inequities faced by older Asian Americans (21).

## Structural Inequities Faced by Older Asian Americans

The media's negative portrayal (e.g., #BoomerRemover) of older adults and discriminatory healthcare practices have reinforced ageist stereotypes that older adult lives are less valuable. The unprovoked targeting of older Asian Americans in anti-Asian hate incidents has placed them at increased risk of physical and emotional harm (22). Fear of going out to public spaces decreases social and health resources (e.g., ethnic grocery stores or seeking care) available to older adults and prevents them from leaving home for regular needs or seeking healthcare (21). The economic downturn in neighborhoods (e.g., Chinatowns) has contributed to social and linguistic isolation (23). Limited policy attention to the social determinants of health among older adults in communities of color, including Asian American communities, create barriers in access to healthcare and social services (17). The cultural norm of familial collectivism has been used to dismiss the need for culturally- and linguistically-appropriate resources for older Asian Americans and their families. The following sections describe how key determinants of health in older Asian American communities—immigration, language and telehealth access, and economic conditions—contribute to COVID-19 risk and barriers to care.

### Older Asian Immigrant Workers in Frontline Industries

About 85% of older Asian Americans are foreign-born, more than any other U.S. racial/ethnic group (10), with the highest rates of foreign-born among South Asian, Vietnamese, Korean, Filipino, and Chinese older adults (Table 1). Asian immigration to the United States has historically been influenced by the demand for a constant labor supply, especially after the 1965 Immigration and Nationality Act and establishment of work visas. This has patterned certain Asian immigrant subpopulations into working in frontline essential occupations with high COVID-19 exposure or inadequate protection (25). About one in five Asian American and Pacific Islander adults aged 55 years and older work in frontline service job, compared to only 15% of the total U.S. population (26).

**Table 1:** Characteristics of Asian American adults aged 55 years and over, American Community Survey 2015–2019^a^.

	**Total Asians^**b**^**	**Total Asians (in Table)^**c**^**	**Chinese**	**Filipino**	**Japanese**	**Korean**	**Vietnamese**	**South Asian^**d**^**
Total population, *n*	21,399,658	16,083,244	4,107,720	2,868,819	770,672	1,464,789	1,815,183	5,066,061
Adults 55+ years, *n*	4,553,178	3,921,418	1,096,468	854,180	343,839	393,150	440,441	793,340
% Adults 55+ years	21.3%	24.4%	26.7%	29.8%	44.6%	26.8%	24.3%	15.7%
**Age groups, years**								
55–64	48.0%	47.2%	47.3%	46.7%	37.1%	47.6%	50.2%	50.0%
65–74	31.6%	31.6%	30.3%	33.4%	29.3%	29.7%	31.9%	33.4%
75–84	14.9%	15.4%	15.5%	15.3%	19.2%	17.8%	13.6%	13.6%
85+	5.5%	5.8%	6.9%	4.6%	14.4%	4.9%	4.4%	3.0%
**Sex**								
Men	44.2%	44.1%	45.5%	38.3%	39.5%	37.7%	46.6%	51.9%
Women	55.8%	56.0%	54.5%	61.7%	60.5%	62.3%	53.4%	48.1%
**Education**								
No school or N/A	7.3%	6.5%	9.0%	2.0%	1.3%	3.8%	14.2%	7.4%
Less than high school	10.7%	11.0%	14.9%	7.3%	3.7%	9.7%	16.5%	10.2%
High school graduate or GED	27.1%	26.8%	25.8%	23.4%	30.6%	34.5%	36.5%	20.8%
Some college	15.8%	15.3%	12.0%	20.9%	22.3%	14.7%	16.1%	10.4%
College degree or more	39.2%	40.4%	38.2%	46.3%	42.2%	37.3%	16.6%	51.2%
**English proficiency**								
Does not speak English or does not speak English well	29.2%	30.3%	45.6%	10.3%	7.0%	41.2%	53.6%	22.5%
Speaks English^e^	70.8%	69.7%	54.4%	89.7%	93.0%	58.8%	46.4%	77.5%
**Citizenship status**								
U.S.-born	15.0%	12.2%	10.8%	9.9%	64.5%	4.3%	3.2%	2.8%
Foreign-born	85.0%	87.8%	89.3%	90.1%	35.5%	95.7%	96.8%	97.2%
**Median annual household income**	$87,126	$88,434	$80,000	$102,914	$87,600	$64,455	$68,396	$1,115,94
**Annual household income**								
Less than $25,000	17.5%	14.8%	19.8%	8.1%	11.8%	23.2%	19.3%	9.4%
$25,000–34,999	8.9%	6.0%	6.4%	4.9%	6.3%	7.6%	7.4%	4.9%
$35,000–49,999	12.5%	8.9%	8.6%	7.7%	9.7%	10.0%	11.1%	8.2%
$50,000–74,999	17.7%	13.7%	12.9%	13.9%	15.5%	14.4%	16.3%	12.1%
$75,000–99,999	12.8%	11.7%	10.5%	13.8%	13.1%	11.0%	12.4%	10.8%
$100,000–149,999	14.8%	17.7%	15.7%	22.0%	19.0%	15.3%	16.1%	17.8%
$150,000 or more	15.8%	27.2%	26.1%	29.6%	24.6%	18.4%	17.5%	36.8%
**Multigenerational household** ^**f**^	17.8%	18.3%	15.9%	20.9%	6.4%	8.1%	20.5%	27.7%
**Essential workers**	23.8%	23.9%	21.1%	28.4%	14.4%	27.0%	28.9%	22.7%
Healthcare	7.6%	7.9%	6.0%	14.8%	3.7%	5.9%	3.8%	8.0%
Food prep services	0.6%	0.5%	0.3%	0.8%	0.5%	0.3%	0.3%	0.7%
Personal care	3.3%	3.4%	5.2%	2.9%	2.1%	3.5%	3.2%	1.8%
Protective services	2.1%	2.2%	1.6%	1.5%	1.6%	2.6%	7.1%	1.2%
Sales	5.5%	5.5%	4.7%	4.3%	5.0%	9.7%	3.1%	7.6%
Production	4.7%	4.5%	3.3%	4.1%	1.6%	5.1%	11.5%	3.4%

a *Data from the American Community Survey 2015–2019 5-years estimates were analyzed (24).*

b *“Total Asians” is from the variable “RACASIAN” in the IPUMs dataset. This includes people who self-reported Asian race and includes other Asian ethnicities not detailed in the table (e.g., Cambodian, Lao, Thai).*

c *“Total Asians (in Table)” includes Chinese, Filipino, Japanese, Korean, Vietnamese, and South Asian groups.*

d *“South Asian” includes people who self-reported being Asian Indian, Bhutanese, Nepalese, Bangladeshi, Burmese, Pakistani, and Sri Lankan.*

e *“Speaks English” includes people who speak only English, speak English very well, and speak English well.*

f *Multigenerational Household is defined as family households consisting of three or more generations*.

There is an alarming rate of COVID-19 infection and deaths among Filipinx Americans (27), who have a high proportion of frontline healthcare workers (15%) relative to other Asian subgroups (Table 1). Filipinx make up 4% of the nursing workforce but comprised 31% of all COVID-19 nursing fatalities as of September 2020 (28). Many Filipinx healthcare workers were recruited to high-risk frontline positions through the establishment of U.S.-style medical schools in the Philippines during the U.S. occupation (25). Older age, high burden of comorbidities, and employment in frontline/essential industries have contributed to disproportionate COVID-19 infection and mortality in Filipinx American healthcare workers (27). Smaller Asian subgroups like Nepalese and Thai adults are also highly represented in frontline/essential industries but there is limited information the COVID-19 impact on these communities (29).

### Economic Disparities Faced by Older Asian Immigrants

Contrary to the public perception of Asian Americans as the socioeconomically successful model minority, older Asian American immigrants are more likely to be poor, have fewer assets and are less likely to own a home and vehicle than older white and Hispanic/Latinx immigrants (30). Older Asian immigrants are economically worse off than their U.S.-born Asian counterparts, and the U.S.-born vs. immigrant-born wealth gap is the largest of any racial/ethnic group (30). Some reasons for this gap are that Asian American immigrants experience financial barriers and discrimination in the labor market. Older Asian immigrants are also susceptible to economic consequences (e.g., business closure) during the pandemic, and may lack generational wealth and the financial ability to bounce back from the decline (23).

Individuals with economic insecurity are at higher risk of infection and adverse consequences of COVID-19 infection, partially due to inability to socially distance because of crowded housing conditions (31). For example, about 66% of Asian older adults living in poverty resided with family, compared to 40% of non-Asian older adults in New York City (32). More than 20% of South Asian, Filipinx, and Vietnamese American older adults live in multigenerational homes (Table 1). Physical and social distancing during the pandemic is especially difficult in crowded housing conditions, where there is increased risk of intra-household transmission of coronavirus (33, 34), especially in households with high-risk older adults, frontline workers, and individuals without insurance. Although the national vaccination guidelines prioritized older adults, only a few states have flexed their plans to include prioritization for household members living with older adults to ensure adequate protection against intra-household COVID-19 transmission (35).

### Threat of Public Charge as a Barrier to Eligible Public Assistance

Many immigrants, including Asian Americans, arriving in the U.S. are often of older age due to long waiting times for visas and have delayed access to public benefits like Medicare due to ineligibility based on citizenship status. The threats of being labeled as a “public charge” or becoming inadmissible for lawful permanent residence (LPR) or citizenship have hindered low-income immigrants including LPR to seek for public benefits and COVID-19 related support (36–38). Despite the recent removal of public charge criteria, fears about losing eligibility for citizenship by using public services persist. The increased xenophobic and anti-immigrant rhetoric has also prevented many permanent immigrants from utilizing public social and healthcare services, like getting COVID-19 tests or vaccination. For example, citizens and LPR were prioritized ahead of undocumented immigrants in Nebraska, despite citizenship not being a requirement for vaccination. Conflicting comments from government officials increases confusion about eligibility for COVID-19 testing and vaccination among immigrants and further strokes fears of public charge, despite the public health need to vaccinate (39).

### Language and Digital Barriers to COVID-19 and Routine Care

Older Asian Americans with diverse language needs and limited digital access have difficulties seeking care in healthcare systems that have not accommodated patients with limited English proficiency (LEP) nor provided age-friendly remote services. Asian Americans include more than 40 ethnicities and 100 different languages and dialects (40). Higher percentages of LEP (not speaking English or not speaking English well) are found in Vietnamese (51%), Chinese (46%), and Korean (41%) adults aged 55 years and older, compared to the average of 29% among all Asians aged 55 and older (Table 1). Having LEP is associated with greater COVID-19 infection risk and presents barriers to accessing health services/insurance and understanding health information, especially when interpreters, culturally and linguistically matched providers, and in-language information are not available (41). Compared to their counterparts who are fluent in English, Asian Americans with LEP are more likely to not have a usual source for care, not have regular check-ups, have unmet medical needs and experience patient-provider communication problems, resulting in underutilization of healthcare services and diminished quality of care (40, 42, 43). For older Asian Americans with LEP and chronic conditions, the linguistic barriers have placed them at a disadvantage and unequal burden of morbidity and mortality.

With the rapid shift to remote and telehealth services during the pandemic, telehealth systems that do not accommodate a variety of languages and technological proficiencies are inaccessible to older Asian Americans with LEP, limited digital access and literacy. Many older Asian Americans who live in ethnic enclaves have substandard broadband Internet access due to historical place-based racism (44). They have been experiencing difficulties in obtaining accurate and timely information in their native language about COVID-19 safety precautions, testing and vaccines; locating testing or vaccination sites; scheduling physician and vaccine appointments; maintaining communication with providers; and applying for public assistance programs that support individuals impacted by the coronavirus (e.g., rental and unemployment assistance) (45). Given these barriers, fewer Asian Americans have been tested and fully vaccinated compared to non-Hispanic whites (25.6 vs. 27%) (46, 47), which potentially lead to greater COVID-19 attributable mortality in older Asian Americans (2).

## Recommendations

We call for an intersectionality-informed approach to public health research, policy and decision making when addressing COVID-19 disparities, and to improve health and well-being of older Asian Americans. An intersectionality framework highlights how power and inequalities differentially impact historically marginalized groups based on their intersecting identities – identifying as an older adult and minoritized group (9).

### Collect and Disaggregate Asian American Health Data

Intersectionality analysis requires race/ethnicity data to be available and disaggregated by subgroup, which is particularly important for the diverse Asian American population (48). “Asian American” and “Native Hawaiians and Other Pacific Islanders” must be collected and reported as two separate racial groups in accordance with federal guidelines (49). Detailed ethnic group data needs to be collected for Asian Americans, and if disaggregated data are not available, there should be explicit explanations to characterize the representativeness of the sample (50). More data is needed especially for Asian subgroups with smaller populations in the U.S. but with greater percentages of working or unemployed older adults (e.g., Sri Lankan, Bangladeshi, Nepalese) or rapidly growing populations in the U.S. (26). Future data collection and reporting should consider the multiple intersecting identities of Asian Americans – age, gender, socioeconomic status, disability, immigration status, sexual orientation and religion. Leveraging innovative data resources, employing both qualitative and quantitative methods, and meaningful inclusion of Asian American subgroups in research could generate more comprehensive data that capture people's lived experiences.

When reporting the disparate effects of COVID-19, older Asian Americans' experiences need to be interpreted in the context of structural inequities, with special consideration to immigration factors, language and digital access, and economic conditions. Of note, the intersecting effects in these structural inequities can look different for various Asian American subgroups. Therefore, centering research on the community from the beginning, building mutually beneficial academic-community partnerships, and engaging communities during the research process can generate findings that are most relevant to the Asian American subgroup experiences.

### Meaningful Inclusion of Older Asian Americans

Intentional inclusion of older Asian Americans and other historically excluded populations in clinical research directly aligns with the National Institutes of Health policies and guidelines (51). Representation of older Asian Americans in clinical trials is necessary to end the longstanding ageist practice of conducting clinical trials with miniscule numbers of older adults and expecting to extrapolate results to be generalizable to all older adults (14). Successful methods of engagement with older Asian Americans would require linguistically- and culturally-relevant resources (i.e., bilingual researchers and materials), partnering with community-based organizations to recruit and retain participants, and using on- and offline modes of information transfer and exchange that are accessible to older Asian Americans (52, 53). In parallel, improving workforce diversity, training, and research with an intersectional lens to understanding health disparities in older adults will promote a more equitable response to advancing health for the aging population (48). Representation and equitable funding for older Asian Americans in clinical research is important because funding provides the needed resources for preliminary research, which determines funding priorities, interventions, and translating research into policy and practices that are equitable (12, 54, 55).

### Investment in Community Initiatives and Uplift Cross-Sector Partnerships

Eliminating structural inequities in determinants of health will require commitment to and investment in Asian American-serving organizations, grassroot efforts, and multisectoral partnerships. Asian American-serving organizations require investments to scale up culturally- and linguistically-concordant resources, such as multilingual helpline and interactive maps, for disseminating COVID-19 vaccine information (47, 56). Training community health workers can help facilitate clinic-community linkages and assistance with clinical and social services (i.e., emergency relief benefits, food pantry programs) (57). Minimizing older adults' barriers to COVID-19 or routine care will require involving family members and bilingual community health workers in care teams, plus multi-sectoral partnerships that can provide transportation and/or internet services for in-person and telehealth visits (58).

We must reconsider the immigration pathways that are heavily linked to essential worker industries and ensure that immigrants have the appropriate occupational health and safety protections. Often times, older Asian immigrants have limited job options and have to work in low-wage, physically-demanding and high exposure industries (25). Relaxing the restrictions for the occupational industries immigrants on work visas can be employed and increasing job opportunities could better support immigrants (59). The U.S. federal government and local administrations could also increase the minimum wage and employee salaries, provide hazard pay, reduce the number of exposure hours at work, and increase paid sick leave for symptomatic or at-risk workers. Similarly, removing barriers related to eligibility for public benefits and rental/home ownership assistance programs could improve the economic security and overall well-being for older immigrant adults. Expanding the vaccine prioritization to include all members living in a multigenerational home could lower the risk of coronavirus transmission in the household (34). We also need to combat ageism at workplace and promote meaningful job opportunities that uplift the agency of older Asian Americans.

The U.S. federal government and local administrations should prioritize collaborations with community-based organizations to protect marginalized populations, including older Asian Americans adults and immigrants. Public health messaging must be clear, timely and consistent in communicating with individuals with limited English proficiency. For example, there should be explicit statements that COVID-19 vaccination and testing is available at no-cost to everyone, regardless of their citizenship status or health insurance (60). Implementing universal healthcare that cover all individuals regardless of immigration and citizenship status could relieve fears related to detention or deportation and increase the national vaccination rate (59). Federal, state, local, and philanthropic resources and funding should be equitable allocated to support multi-pronged and multi-level approaches to meet the needs of diverse older Asian Americans (12, 47, 61), and will require commitment, action and accountability to advance health equity.

## Conclusion

The scenarios presented are not insurmountable but will require innovative reimagining of the public health infrastructure to address health disparities during the COVID-19 pandemic and in the future. This article focused on older Asian Americans and we acknowledge that there is overlap with the experiences of other older adults of color (7). The recommendations present immediate and long-term measures that can mitigate existing disparities and advance a health equity agenda for Asian American communities and other historically marginalized groups, including Black, Indigenous, Hispanic/Latinx, Native Hawaiian, and Pacific Islander older adults.

## Data Availability Statement

Publicly available datasets were analyzed in this study. This data can be found here: Ruggles et al. (24).

## Author Contributions

KM, AB, and LÐ contributed to the conceptualization, drafting and editing of the manuscript. All authors provided critical feedback and helped shape the final version of the manuscript.

## Author Disclaimer

The contents of this publication are solely the responsibility of the authors and do not necessarily represent the official views of the funders.

## Conflict of Interest

The authors declare that the research was conducted in the absence of any commercial or financial relationships that could be construed as a potential conflict of interest.

## Publisher's Note

All claims expressed in this article are solely those of the authors and do not necessarily represent those of their affiliated organizations, or those of the publisher, the editors and the reviewers. Any product that may be evaluated in this article, or claim that may be made by its manufacturer, is not guaranteed or endorsed by the publisher.
